# The role of visa class in the location choices of immigrants in Australia at the regional and neighbourhood scales

**DOI:** 10.1007/s12546-022-09280-w

**Published:** 2022-02-02

**Authors:** Dagmara Laukova, Aude Bernard, Toan Nguyen, Thomas Sigler

**Affiliations:** Queensland Centre for Population Research, School of Earth and Environmental Sciences, The University of Queensland, Brisbane, QLD Australia

**Keywords:** Location choices, Immigration, Settlement patterns, Ethnic networks, Visa class

## Abstract

Australia’s pro-immigration policies have played a vital role in national population growth, serving to address what would otherwise be chronic labour shortages and population ageing. While migrants to Australian have shown a clear preference for cities and tend to locate with co-ethnics, variations by visa class—employment, family reunification, and asylum—have yet to be fully explored. This paper aims to identify variations in settlement patterns of immigrants in Australia by visa types and the factors underpinning these choices, paying particular attention to ethnic networks and employment opportunities. We apply a series of negative binomial regressions to aggregate census data linked to visa status. At the suburb level, our results show the importance of the presence of compatriots in shaping the location choices of family migrants, with the exception of skilled and humanitarian immigrants from China, Malaysia and Thailand. At the regional level, skilled migrants, including skilled regional migrants, respond to employment opportunities to a greater extent than family and humanitarian migrants.

## Introduction

With almost a third of its population born overseas (ABS, 2017), Australia is a high immigration country by global standards. Since settlement of Australia began, migration has played a major role in shaping the size, composition, and distribution of the population. While immigration was initially a means to further colonial aspirations on the vast continent, its role has evolved over time to become a vehicle for national and regional economic growth by addressing skill shortages and moderating the pace and extent of population ageing (McDonald, 2017). The beginning of the twenty-first century stands out as the period with the highest levels of net overseas migration since World War II.

In that context, understanding the location choices of immigrants is of growing importance at both the macro and micro levels. At the population level, the settlement patterns of overseas-born migrants exert a significant effect on the growth, composition, and geographic distribution of Australia’s population (Bell & Hugo, 2000; Raymer & Baffour, 2018). Understanding of such processes is essential for planning purposes and services provision as spatial imbalances can reinforce regional socio-economic disparities (Gutiérrez et al., 2018). At an individual level, spatial integration is an important component of the settlement process (Massey & Denton, 1985; Wessel et al., 2017) since the place of residence can reinforce or mitigate the socio-economic imbalance of disadvantaged groups by constraining or facilitating access to services, social networks, and employment.

Migrants’ location choices are a very complex process whose understanding is complicated by the fact that migrants form a heterogeneous group, not only with respect to the country of origin but also reasons for moving to Australia. Some enter the country for employment reasons, while others look to reunite with family members and others seek refuge. There are also differences between those who aim to migrate permanently and those who come temporarily, for example university students. In Australia, humanitarian migrants do not appear to follow conventional settlement drivers since they often participate in special government assistance programs (Hugo, 2002), whereas temporary skilled migrants are highly concentrated in state capitals and their settlement patterns tend to resemble those of the native-born population (Hugo, 2004). However, there has been no comparative research on location preferences of migrants from various visa classes and thus there has been no systematic approach to compare the location choices of migrants who have settled in Australia on different visas. As a result, the extent to which migrants differ in their settlement choices depending on their visa class remains largely unknown. Evidence from the Netherlands suggests that humanitarian migrants are a spatially diffused group on first arrival but over time tend to move to more segregated neighbourhoods and are less responsive to economic conditions, which stands in stark contrast to other migrant groups, particularly labour migrants (Zorlu & Mulder, 2008).

It is therefore reasonable to assume that reasons for immigrating to Australia bear some influence on the choice of place of settlement. For instance, skilled migrants’ location choices are more likely to be shaped by economic considerations and employment opportunities whereas family migrants, who move to re-unite with family members already established in Australia, may be less responsive to these factors. On the other hand, access to ethnic networks is likely to play a greater role in the settlement decisions of humanitarian migrants who have less access to material resources upon arrival and may be more reliant on assistance of various kinds by those speaking the same language, among other common attributes. Understanding such settlement patterns is complicated by the fact that some visas such as those from the skilled regional migration scheme require the recipients to reside in a particular regional area for a minimum of two years. These visa requirements are likely to play out more at a labour market rather than at the residential level, where ethnic networks are more likely to operate. However, the impacts of these spatial scales are likely to interact to affect location choices of immigrants by generating broad settlement patterns.

The recent release of the linked visa status and census dataset, the Australian Census and Migrants Integrated Dataset (ACMID), presents an unprecedented opportunity to empirically examine how migrants who settled in Australia under different visa streams respond to ethnic networks and economic factors in their location choices. ACMID encompasses all permanent migrants that have been granted permanent residency in Australia since the 1st of January 2000 via one of the three visa classes, skilled, family and humanitarian, and who were still residing in Australia at the 2016 census, which corresponds to 2.16 million individuals.

Drawing on this novel aggregate dataset and using a series of negative binomial regressions, this paper aims to provide a more fine-grained understanding of the factors underpinning contemporary patterns of immigrant settlement. The work extends our knowledge of the determinants of settlement in two principal ways. First, it aims to gain insights into location choices by recognising heterogeneity within the migrant population and allowing the determinants of location choices to vary based on visa type and country of origin. Second, the study examines settlement choices at both regional (SA4) and suburban level (SA2) in order to understand how the mix of factors determining location choices varies depending on the geographical level of analysis. In doing so, we attempt to bridge the gap between, on the one hand, economic and geographic approaches, which favour analysis at a regional or labour market level with emphasis on the role of economic opportunities and, on the other hand, sociological perspectives, which focus on residential segregation and the socio-demographic factors at the suburban level. By addressing these questions, we seek to create a robust and comprehensive evidence base to aid policy development and advance theorisation of settlement processes.

The paper begins with an overview of the immigration program and policies in Australia in ‘Australian migration in context’ and ‘Understanding Australian migration through visa class’. ‘Conceptual framework’ reviews theoretical and empirical approaches to understanding the settlement patterns of immigrants in Australian and beyond. ‘Data and methods’ introduces the ACMID dataset and presents the regression models employed. ‘Descriptive statistics’ reports descriptive results by spatial scale, visa class and country of birth in the form of tables compared to the settlement patterns of Australia-born population before presenting results from our regression analysis in ‘Drivers of settlement’. The last section concludes by discussing the theoretical and policy implications of our results and proposing avenues for future research.

## Australian migration in context

**Fig. 1:**
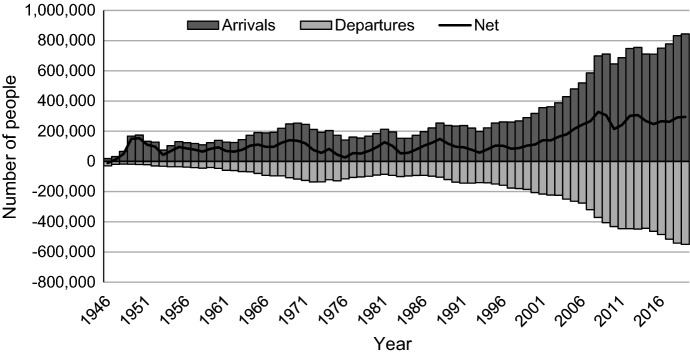
Permanent and long-term overseas arrivals and departures in Australia 1946–2019. *Source* Authors’ calculations based on ABS 2019 catalogue number 3401.0

From 2013 to 2019, the annual net overseas migration intake hovered around 300,000 (Fig. 1), contributing to about 60% of the national population growth (ABS, 2019a, b). As a result, the rate of population growth in 2019 was five times higher than the average rate in more developed regions (UN, 2019) and the fifth highest rate among OECD countries (World Bank, 2020). While Australia’s net overseas migration turned negative in June 2020 (with 5900 more people departing than arriving) due to the international border closure in response to the COVID-19 crisis, this is expected to be a temporary drop followed by a return to long-term trends by 2027–28 (Centre for Population, 2020).

The beginning of the twenty-first century has seen not only a substantial growth in the level of net overseas migration to Australia (ABS, 2019a, b), but also an increase in the share of skilled migrants from 32% in 1997 to more than 60% in 2018. These changes have occurred in a context of diversification of origin countries in favour of Asia (Jupp, 2001; Raymer et al., 2018, 2020; Wilson & Raymer, 2017). In 2014, China and India became for the first time the top two origin countries in terms of net annual flows, indicating the relative importance of migration from Asia having overtaken that from Europe (ABS, 2018). The process of diversification started gradually in the early 1970s when Australia scrapped its discriminatory white immigration policy and opened its borders to non-European migrants. Since then, the stock of immigrants born in Asia has been growing steadily and in 2016 those born in China were the third largest group of foreign-born people after the UK and New Zealand, with another five Asian countries in the top ten list of the overseas-born as shown in Table 1. While the proportion of China and India-born of the total overseas-born population was 3.5 and 2.3% respectively in 2001, at census 2016 it rose to 8.3 and 7.4%. The dominant position of migrants from Asia is apparent not only in the stock of migrants but also in the annual net overseas migration flows. In 2016 migrants from China and India represented 20 and 18% of the total net overseas migration.

**Table 1:** Top 10 countries of origin in Australia 2001–2016 (total numbers and proportions calculated of total overseas-born population), stock data

Census 2001			Census 2006			Census 2011			Census 2016		
Country	Total	%	Country	Total	%	Country	Total	%	Country	Total	%
UK	1,036,261	25.2	UK	1,038,163	23.6	UK	1,098,709	20.8	UK	1,087,756	17.7
New Zealand	355,762	8.7	New Zealand	389,465	8.8	New Zealand	483,398	9.2	New Zealand	518,462	8.4
Italy	218,722	5.3	**China**	**206,588**	**4.7**	**China**	**318,969**	**6**	**China**	**509,558**	**8.3**
Vietnam	154,818	3.8	Italy	199,124	4.5	**India**	**295,362**	**5.6**	**India**	**455,385**	**7.4**
**China**	**142,807**	**3.5**	Vietnam	159,850	3.6	Italy	185,402	3.5	Philippines	232,391	3.8
Greece	116,431	2.8	**India**	**147,106**	**3.3**	Vietnam	185,039	3.5	Vietnam	219,351	3.6
Germany	108,214	2.6	Philippines	120,540	2.7	Philippines	171,233	3.2	Italy	174,042	2.8
Philippines	103,915	2.5	Greece	109,990	2.5	South Africa	145,683	2.8	South Africa	162,450	2.6
**India**	**95,445**	**2.3**	Germany	106,524	2.4	Malaysia	116,196	2.2	Malaysia	138,363	2.2
Netherlands	83,290	2.0	South Africa	104,132	2.4	Germany	108,002	2.0	Sri Lanka	109,850	1.8

*Source* Author’s calculation based on Census data, 2001, 2006, 2011, 2016. Countries are ranked in descending order in each census. China’s and India’s figures are in bold to highlight their rapid growth

In Australia, migrants’ marked preference for residing in large metropolitan areas, particularly Sydney and Melbourne, has been explained by the educational and economic opportunities that cities offer (Hugo, 2008a) combined with access to socio ethnic networks (Forrest et al., 2006) that result from successive migration waves and facilitate access to resources upon arrival. While ethnic segregation in Australia is lower than in other countries (Forrest & Poulsen, 2003), some groups particularly many from Asia stay closer to their compatriots while others, especially northwest European migrants, tend to follow patterns of settlement close to the Australian population (Hugo, 2011). In addition, Chinese and Indian migrants are predominantly settled in Sydney and Melbourne, whereas migrants from New Zealand and Oceania are overrepresented in Brisbane. On the other hand, Filipino migrants make up a significant proportion of overseas-born in the Northern Territory, while Zimbabweans are highly concentrated in Western Australia, and migrants from Europe and South Africa constitute a high proportion of immigrants in its state capital of Perth (Edgar & Lucas, 2016; Le, 2008; Raymer et al., 2020). Accordingly, the residential geographies of each major city demonstrate unique patterns, yet certain patterns emerge through a deeper analysis based on visa class.

## Understanding Australian migration through visa class

Australia has a long history of migration, and its immigration system is one of the most highly planned and managed in the world (Hugo, 2014b). Based on length of stay, there are two main visa categories—permanent and temporary. All permanent visas allow holders to stay and work in Australia indefinitely and to apply for citizenship after a minimum of four years of residence. There are three main permanent visa classes—skilled, family and humanitarian. For the purpose of this project, we have distinguished an additional visa class—skilled regional visa, by grouping together all skilled visa subclasses with a regional aspect. Applicants can apply for a permanent visa directly offshore or in a two-step process onshore when they first enter Australia on one of the eligible temporary visa programs (temporary work visa, student visa) and after a defined period of time they can apply for a permanent visa. While very common, visa transitions are not available in cross-sectional datasets such as ACMID.

**Fig. 2:**
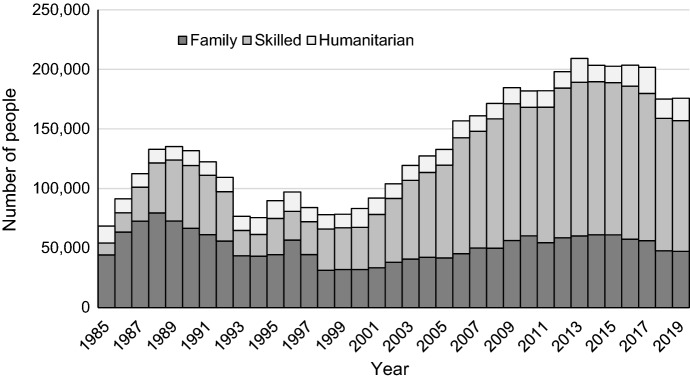
Permanent visas granted in Australia 1985–2019 by visa class. *Source* Authors’ calculations based on Home Affairs 2020

Over time, the intensive skill targeting has progressively changed the visa composition of migrants in Australia. While in the post-war immigration wave, family visas dominated the immigration program, in the mid-1990s the skilled stream took over family reunions (Larsen, 2013) as a result of direct government strategy to meet the needs of the labour market and achieve greater economic gains from immigration (Phillips & Spinks, 2012). Skilled migration now accounts for 62% of all permanent migrants compared with 26% of family migrants as shown in Fig. 2 (ABS, 2019a, b).

While initially Australian immigration policy was mainly concerned with shaping the level and composition of the immigrant intake, since the mid-1990s, the government increasingly attempted to influence where immigrants settle after arriving in Australia (Hugo, 2011). The rapid growth of metropolitan areas and increase in skill shortages in regional areas have led to calls to redirect immigrants toward regional areas. As a response, in 1997, a series of new permanent skilled visas under the State Specific and Regional Migration scheme was introduced. Their aim was to integrate immigration with regional planning allowing states and territories to be more involved in the migration process (Akbari & MacDonald, 2014). Today regional visa holders are still required to reside in a designated regional area for a minimum of 2 to 3 years based on a particular visa type. However, after the initial compulsory regional settlement, migrants are free to move anywhere in Australia and thus the efficacy of these schemes is limited (Guan, 2020; Hugo, 2008b; Wulff & Dharmalingam, 2008). Over the years, the regional aspect has been incorporated in other visa streams as well. For example, humanitarian migrants without family ties in Australia are increasingly being placed in regional areas with adequate services and employment opportunities (Sypek et al., 2008) and work and holiday makers are incentivised to work in regional areas to prolong their stay in Australia (Hugo, 2008b). So far, there has been no systematic evaluation of the regional migration initiatives but the highest growth in employment remains in cities not in regional areas, the former attracting the majority of immigrants (McDonald, 2017).

## Conceptual framework

Settlement patterns of immigrants can vary from complete spatial assimilation in which migrants’ residential geographies ultimately mirror those of the host society (Fong & Wilkes, 1999; Gordon, 1964; Massey & Denton, 1985) to complete segregation whereby they are isolated in particular suburbs (Frey, 1980; Frey & Liaw, 1998). Nonetheless, the reality is far more complex than this simple dichotomy and lies somewhere in between in most instances, with most studies pointing to complex and varied settlement patterns shaped by socio-economic characteristics, ethnicity and time spent in the host country.

Two main theories explain the different settlement outcomes: spatial assimilation and network theory. The spatial assimilation perspective, derived from assimilation theory, which was first formulated in the first half of the twentieth century (Bogardus, 1933; Burgess, 1947; Gordon, 1964; Park, 1926), assumes that immigrants first reside in ethnically segregated neighbourhoods before relocating to areas predominantly occupied by natives as the duration of their residence increases. This suggests that economic motives may take over social ones as migrants become more established (Glick & Park, 2016), although the pace at which spatial assimilation occurs depends also on age at arrival, country of birth and socio-economic status of migrants (Burgess, 1947; Park, 1926). Empirical evidence from Australia indicates that the settlement patterns of the overseas-born broadly converge to those of natives within a decade (Bell & Cooper, 1995; Guan, 2020).

The spatial assimilation approach has been widely applied in North America (Ellis et al., 2006; Fong & Wilkes, 1999; Newbold & Spindler, 2001), where empirical studies have focused on the neighbourhood scale of settlement by ethnicity or countries of birth of immigrants. In Canada, Fong and Wilkes (1999) concluded that some European immigrants and their descendants have mostly spatially assimilated whereas particular Asian migrants’ settlement patterns do not relate to their socioeconomic status because of the role of ethnic networks in shaping their location choices, particularly upon arrival. In United States, Ellis et al. (2006) showed the importance of household composition in the process of assimilation or segregation, with migrants with partners from another country being less likely to live in segregated neighbourhoods.

The process of spatial assimilation has since been examined in countries around the world (Andersen, 2010; Burgers & van Der Lugt, 2006; Edgar, 2014; Janská et al., 2013; Macpherson & Strömgren, 2013; Wessel et al., 2017; Zorlu & Mulder, 2008). For example in the Netherlands, migrants’ patterns of settlement appear to be close to that of the Dutch population (Burgers & van Der Lugt, 2006), although there seem to be important variations depending on motives for immigration, employment, family reunification, family formation and asylum (Zorlu & Mulder, 2008), with humanitarian migrants showing the most distinctive settlement and internal migration patterns by being the least sensitive to economic conditions and most prone to move to segregated neighbourhoods.

In light of the varied settlement patterns of immigrants, scholars have progressively moved away from the idea of uniformed assimilation to recognise the plurality of lived experiences. This is best encapsulated in the theory of segmented assimilation, which seeks to capture and understand the nuances of migrants’ integration (Portes & Zhou, 1993) and the wide spectrum of settlement patterns. This perspective recognises that immigrants spatially cluster upon arrival based on their socio-economic characteristics such that multiple assimilation processes happen but at a different pace for different migrant groups. Most empirical studies on segmented assimilation have focused on residential segregation (Edgar, 2014; Forrest et al., 2006; Poulsen & Johnston, 2000; Wang et al., 2018). These studies have shown in the Australian context that Chinese migrants, for example, have formed multiple distinct enclaves with different levels of spatial assimilation based on educational level, English proficiency and income (Wang et al., 2018), with Mainland China-born found to be stronger in big cities (Sydney, Melbourne and Brisbane) while Taiwan-born are more segregated in medium and smaller-size cities (Hobart, Adelaide, Darwin, Canberra).

As a result, there has been a growing interest in understanding the determinants of settlement patterns, both in the Australian context and globally (Chiswick et al., 2001; Maza et al., 2013; Zavodny, 1999; Zorlu & Mulder, 2008). The determinants of migration have commonly been formulated in the context of individual utility maximisation, although in recent years increasing emphasis has been placed on the family or the household as the decision-making unit (Bushin, 2009; Cooke, 2008; Perales & Vidal, 2013). Globally, the most accepted determinant of the location choices of immigrants is the presence of previous immigrants, supporting the network migration theory (Bartel, 1989; Dunlevy, 1991; Zavodny, 1999). A recent Spanish study revealed that employment rate and the stock of established migrants are the most important determinants of location choice of migrants (Viñuela et al., 2019).

The persistence of differentiated patterns of migrant settlement over time suggests that co-ethnic communities play an enduring role. This can be explained through the lens of migration network theories (Durand & Massey, 1992; Massey et al., 1993) on perpetuation of international migration, which emphasises the role of diasporic networks in maintaining segregated patterns of settlement for some immigrant groups. Network theory originates in the theory of chain migration (MacDonald & MacDonald, 1964) in which new immigrants settle in close proximity to relatives or friends who provide beforehand information and support upon arrival including transport, accommodation or even employment. Networks not only facilitate migration, but also encourage settlement in particular locations with a high proportion of compatriots who facilitate adjustment in a new location by providing access to local resources. It is the case in Sydney and Melbourne, which have long been gateways (Hugo, 2008a) to Australia because of strong existing networks of overseas-born migrants from different nations. Disaggregation by country of birth reveals distinct patterns of migrant settlement in different suburbs.

While there is a large body of literature on the internal migration of immigrants in Australia (Bell & Cooper, 1995; Bell & Hugo, 2000; Hugo, 1983, 2002, 2008b; Raymer & Baffour, 2018; Raymer et al., 2018), only few studies have explored the role of ethnic networks (Chiswick et al., 2001; Wilson, 1994; Wulff & Dharmalingam, 2008), focusing instead on age at migration, duration of stay in Australia and English proficiency. We add to this literature by taking into account visa types and exploring how they interact with country of birth to generate particular patterns of settlement. The strictly controlled immigration system in Australia offers a unique opportunity to empirically determine the effect visas have on locational choices of immigrants. While some descriptive studies have considered the role of visa class in the settlement and migration processes, they have focused on one specific visa class (Davern et al., 2016; Hugo, 2014a; Hugo et al., 2003; Khoo et al., 2008; Larsen, 2013) or on one particular ethnic group (Chiang & Hsu, 2005). Thus, there has been no systematic analysis of settlement drivers of immigrants in Australia by visa class.

## Data and methods

To quantify the roles of ethnic networks and economic drivers on the location choice of immigrants that settled in Australia on different visa classes, we use cross-sectional data, a census linked dataset, The Australian Census and Migrants Integrated Dataset (ACMID), that provides a wide range of migrants’ characteristics including visa class category. The ACMID probabilistically matches individual-level records from the 2016 census to the visa status of immigrants acquired from the Department of Immigration and Border Protection’s Settlement Database. ACMID represents the stock of all immigrants who received a permanent residency visa via the skilled, family or humanitarian stream any time after 1 January 2000 and were still living in Australia at the 2016 census. For confidentiality purposes, the data have been released publicly only at an aggregate level. The data set is publicly available through the Australian Bureau of Statistics online tool—TableBuilder that is used to disseminate aggregate level census data.

**Fig. 3:**
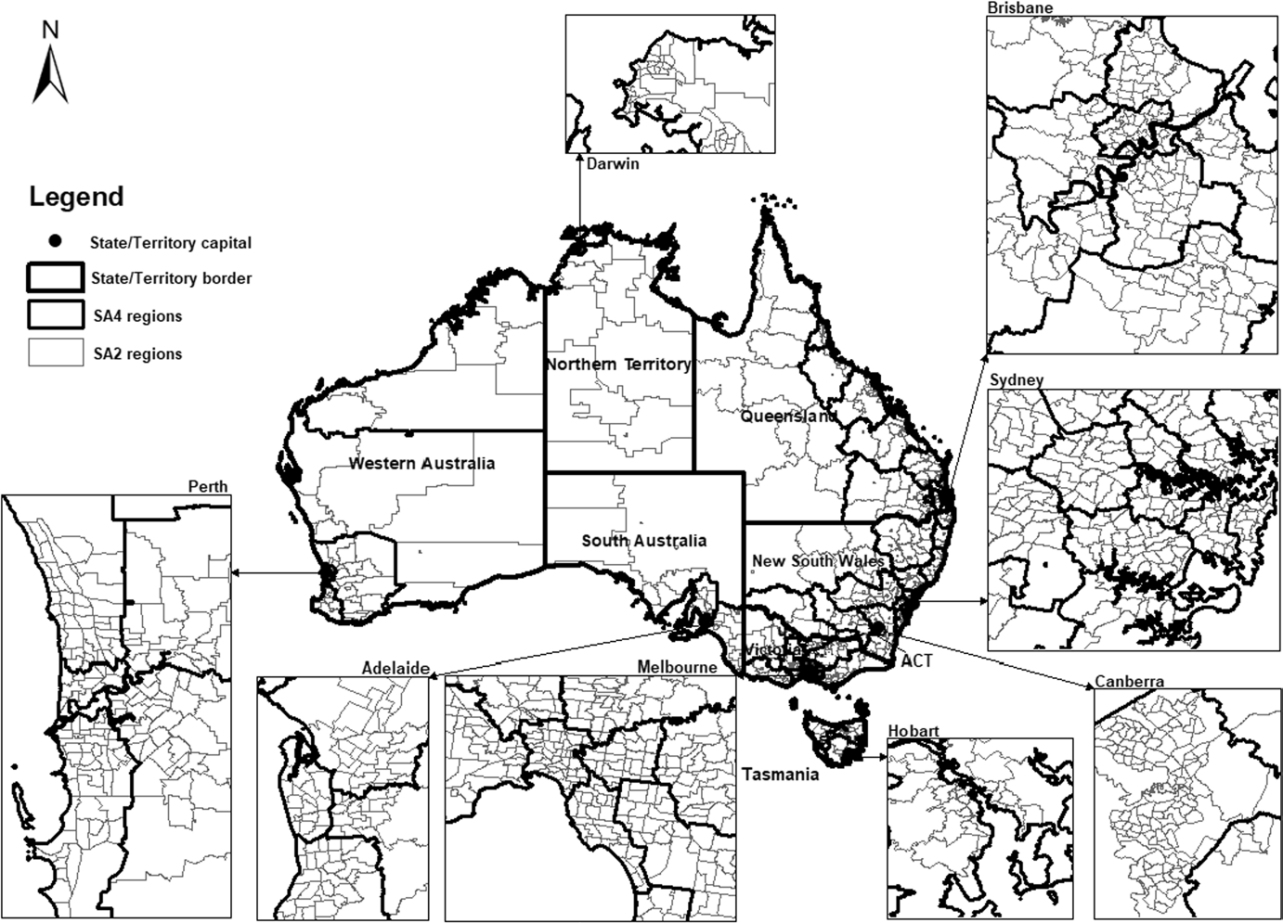
Spatial units in Australia (States/Territories, SA4 and SA2 regions). *Source* Produced by authors using ArcGIS

To capture settlement patterns at both the local and regional level, we calculate all our variables at two spatial levels—Statistical Area Level 4 (SA4), the largest sub-state regions which broadly correspond to labour markets, and Statistical Area Level 2 (SA2), which correspond to residential suburbs as shown in Fig. 3. There are 87 SA4 regions and 2,196 SA2 units, which are the most suitable spatial units for analysis of ethnic networks because of the strong interaction between people that typically happens at this level.^1^

To establish the determinants of settlement patterns of migrants we use total count of immigrants in each SA4 and SA2 (dependent variable) by visa type and country of birth. We consider four pathways to permanent residency represented by the following visa streams -skilled, skilled regional, family and humanitarian. Because the Australian census does not collect information on ethnic background, we use country of birth as a proxy for ethnicity. We consider ten countries of birth: India, China, Malaysia, Sri Lanka, Pakistan, Thailand, Iran, Zimbabwe, Nepal, and Egypt, all of which are well represented by higher numbers of migrants in each visa class, including humanitarian entrants, to ensure comparability across visa types for the same countries. For this reason, some of the most populous migrant groups (UK, New Zealand) are not included in this paper because they are not represented in the humanitarian category.

Our estimation framework for Negative Binomial Regression model is as follows:

1 $${NI}_{r,c,v}={ {\alpha }_{r}+ \beta }{X}_{r,c-5}+ \gamma {Z}_{r-5}+ {\varepsilon }_{r,c,v}$$

where the dependent variable $NI$ is the number of immigrants from country *c* in region *r* on visa class *v*, ${X}_{r,c-5}$ are explanatory variables pertaining to each region *r* and country of origin *c* (proportion of foreign-born and proportion of compatriots), ${Z}_{r-5}$ are variables that relate to regions, namely population size, median age, proportion of homeowners, median weekly household income, proportion of tertiary educated, unemployment rate and share of public housing. We use the log of continuous variables expressed as counts (population size, income, and median age) to facilitate interpretation as a percentage change in the dependent variable. To capture variations by visa and country of origin we add a series of interaction terms between visa class and proportion of foreign-born/unemployment rate, and country of birth and proportion of compatriots/unemployment rate. All covariates are lagged by 5 years and drawn from the previous census (2011) to more realistically represent the information on which immigrants based their decision to settle or move to a particular location. All models are implemented using negative binomial regression, with standard errors clustered by SA4 regions to account for possible interdependences within the regions.

We follow a three step approach in our modelling. First, we focus on a comparison of permanent migrants and Australian-born, then we differentiate immigrants by visa types, and lastly, we distinguish them also by country of birth. In model 1, we run a regression model with Australian-born and all permanent migrants regardless of the visa type as dependent variables to compare settlement patterns of immigrants with that of the natives. The Australian-born population is the reference category. We run the regression on both regional (SA4) and local (SA2) levels (models 1a, 1b). Then to capture the variations between visa classes, we restrict the analysis to migrants, using family migrants as the reference category, and again we repeat the same model on SA4 and SA2 levels (model 2a, 2b). Further, to establish differences among countries of origin, we run a separate regression for each country on both spatial scales distinguishing between different visa types and using family migrants as a reference category, models 3a–j (*n* = 348 (87 SA4s × visa type) and models 4a–j (*n* = 6,336 (2,112 SA2s × visa type). The skilled regional visa data are only available for six out of the ten selected countries (India, China, Malaysia, Nepal, Sri Lanka, and Zimbabwe) and at the SA2 level they are not available for any country due to data sparsity.

## Descriptive statistics

To establish immigrants’ patterns of spatial concentration, Table 2 displays the Index of Dissimilarity^2^ (ID) by visa class and country of birth at regional (SA4) and suburban (SA2) levels. It can be interpreted as the proportion of a migrant group that would have to move out of a spatial unit in order to mirror the distribution of the Australia-born population. Index levels below 20 indicate very little segregation, over 30 significant segregation and index levels above 50 mean extreme segregation. For comprehensiveness and spatial visualisation, Appendix includes a series of maps with settlement patterns of different visa types at the SA4 level (Appendix Figs. 4, 5, 6, 7 and 8).

**Table 2:** Index of dissimilarity by permanent visa types and country of birth in 2016 (SA4 and SA2 level)

	SA4 level						SA2 level				
	All visa	Skilled	Skilled regional		Family	Humanitarian	All visa	Skilled	Skilled regional	Family	Humanitarian
All migrants	31.9	36.2		33.7	31.1	49.0	39.0	43.2	43.2	36.7	63.7
India	42.2	44.8		38.6	44.7	55.8	55.1	55.0	na	57.4	95.6
China	50.3	53.5		41.3	50.4	61.7	60.0	62.0	na	59.0	82.6
Malaysia	47.6	50.2		48.7	42.3	67.6	58.8	61.7	na	81.6	67.5
Sri Lanka	52.0	50.2		49.6	57.7	65.2	61.6	60.9	na	62.8	93.6
Pakistan	49.4	47.7		na	54.4	58.3	63.0	63.8	na	74.2	84.4
Thailand	25.4	39.0		na	21.5	65.9	38.3	68.9	na	34.2	95.5
Iran	42.6	45.3		na	44.7	46.3	56.9	64.3	na	41.8	66.1
Nepal	47.1	54.3		41.4	50.2	78.2	66.8	72.1	na	74.0	84.2
Egypt	45.8	43.2		na	50.8	54.5	64.6	62.5	na	55.4	81.2
Zimbabwe	32.5	36.5		43.5	28.7	41.3	48.0	66.3	na	73.4	86.3

*Source* Authors’ calculation based on ACMID 2016

Family visa holders are the most spatially integrated group, yet the dissimilarity index is over 30. The majority of family migrants join already established migrants or domestic residents and as a result they are mostly concentrated in capital cities, especially in Sydney and Melbourne but they are also represented in regional parts of Australia mainly in North-east Queensland and the Western Australia Outback. The second most spatially assimilated group are skilled migrants, particularly those in regional areas. Skilled regional migrants typically locate in well-established small regional communities and thus, they are more likely to spatially integrate but they have distinctive settlement patterns. Contrary to other migrant groups, they are mostly concentrated in the regional areas of Western Australia Outback, the Northern Territory and Queensland and underrepresented in metropolitan areas of Sydney and Melbourne. They are also highly represented in states’ capital cites classified as regional areas (Canberra, Adelaide, Darwin), and Perth where they are linked to the fly-in fly-out workforce. On the other hand, non-regional skilled migrants predominantly reside in metropolitan areas (Sydney, Melbourne, Brisbane and Perth). Humanitarian migrants are the least spatially integrated group reaching extreme levels of spatial segregation (ID > 50) and predominantly located in states’ capitals (Sydney, Melbourne and Brisbane) and in a few regional clusters (Coffs Harbour, Cairns, Townsville).

Differences by visa types are overlaid by variation by country of origin, with immigrants from Thailand being the most spatially integrated and those from Sri Lanka the least. The general cross-visa pattern holds for most countries of birth. Humanitarian migrants are the most spatially segregated group, with the ID ranging from 41.3 for Zimbabwe, up to 78.2 for Nepal. In all countries but Zimbabwe, skilled regional migrants are less spatially segregated than other skilled migrants. There is, however, some cross-country variations with respect to skilled migrants. Sri Lankan, Pakistani and Egyptian skilled migrants tend to be less spatially segregated than family migrants, while the reverse characterises other countries.

At the suburban SA2 level, the ID increases for all visa classes and countries of birth, but the patterns of spatial segregation by visa types hold, with family migrants being the most spatially integrated visa group and humanitarian migrants being the most segregated. However, six out of ten countries record a marginally lower ID for skilled than family migrants, namely India, Malaysia, Sri Lanka, Pakistan, Nepal, and Zimbabwe, which suggests that their settlement patterns are closer to the Australia-born population. Despite being the most integrated visa group, family migrants still show significant to extreme segregation for all selected countries, except for Thai family migrants. Humanitarian migrants recorded the lowest level of spatial integration for all countries of origin, except for Malaysia, where family migrants show even higher levels. In a few cases the levels reveal almost complete segregation with dissimilarity index over 90 (India, Thailand, Sri Lanka).

## Drivers of settlement

To determine the drivers of location choices of immigrants in Australia, we now report results from regression models. The first set of models (Model 1a–b) uses the Australia-born population as the reference category and does not distinguish migrants by country of birth or visa type (Appendix Table 6). The results indicate that, at both spatial scales, immigrants are more likely to locate in areas with a higher proportion of foreign-born and a lower unemployment rate. Unsurprisingly, while the association with the proportion of foreign-born is stronger at the suburban level, the association with unemployment is higher at the labour market level.

We now examine variations between visa classes, using family migrants as the reference category and excluding the Australia-born population. Regression estimates in Table 3 show that, compared with family migrants, skilled and skilled regional migrants are more likely to locate in areas with a lower unemployment rate, particularly at an SA4 level, whereas the reverse characterises humanitarian migrants at the suburban level.

**Table 3:** Determinants of settlement choices of permanent migrants in Australia in 2016 on regional (SA4) and local (SA2) level, regression estimates

Variables	All permanent migrants	
	SA4 (2a)	SA2 (2b)
Population size in 2011 (log)	1.042***	1.002***
% of owned dwellings	0.009	− 0.002
% of public housing dwellings	0.009	0.012
% of people with higher education (bachelor and higher)	− 0.006	− 0.001
Median weekly household income (log)	− 0.548	− 0.308
Median age (log)	− 3.123***	− 2.561***
Unemployment rate (%)	− 0.044	− 0.079***
Family # unemployment rate (reference category)		
Skilled # unemployment rate	− 0.124***	− 0.103***
Skilled regional # unemployment rate	− 0.154**	− 0.089***
Humanitarian # unemployment rate	0.162	0.168***
% of foreign-born	0.057***	0.050***
Family # % foreign-born (reference category)		
Skilled # % foreign-born	0.028***	0.027***
Skilled regional # % foreign-born	− 0.054***	− 0.019***
Humanitarian # % foreign-born	− 0.005	0.018**
Constant	9.406**	7.130***
Observations	348	8,448
Pseudo R-squared	0.0906	0.102
Log Lik	− 3045	− 46,467

Negative Binomial Regression, cluster by SA4 region robust error. *Source* ACMID 2016, Census 2011

***p < 0.01, **p < 0.05, *p < 0.1

Skilled visa holders are more likely to locate in SA4 and SA2 regions with a higher proportion of overseas-born than family migrants, while skilled regional migrants display the reverse pattern. For humanitarian migrants, the presence of the foreign-born is associated with their locational choices only at the suburban level.

We now seek to establish whether the variations by visa type hold when controlling for country of birth. To that end, we run separate regressions for each country of birth. This also allows us to explore the effect of co-ethnic networks. Results at an SA4 level in Table 4 suggest that broad cross-visa variations hold for most countries of birth notwithstanding subtle variations. With the exception of Sri Lanka, skilled migrants are more likely to settle in regions with lower unemployment, than family migrants and the association is the strongest for migrants from Egypt, China, and Malaysia. While humanitarian visa holders in general seem not to respond to unemployment levels at the regional level, exceptions of strong positive association can be found when disaggregating by country of origin. Migrants from Nepal, Malaysia, Iran, and Pakistan tend to locate in regions with higher unemployment.

**Table 4:** Determinants of settlement choices of permanent migrants by country of birth in Australia in 2016 on SA4 level, regression estimates

Variables	India (3a)	China (3b)	Malaysia (3c)	Sri Lanka (3d)	Pakistan (3e)	Iran (3f)	Thailand (3 g)	Egypt (3 h)	Nepal (3i)	Zimbabwe (3j)
Population 2011 (log)	1.244***	1.582***	1.391***	1.078***	1.183***	1.313***	1.185***	1.237***	0.731**	1.123***
% of owned dwellings	0.018*	0.049***	0.028**	0.015	0.044***	0.046***	0.023*	0.058***	0.041**	− 0.002
% of public housing	0.033	0.053*	0.015	0.003	0.059	0.016	− 0.011	0.062	0.083	0.001
% of people with higher education (Bc and higher)	− 0.004	0.026***	− 0.000	0.001	0.025***	0.038***	0.004	0.026***	0.008	− 0.012***
Median weekly household income (log)	0.725	1.185**	− 1.095	− 0.648	− 2.421***	− 1.020	− 0.691	− 1.554**	− 0.663	0.288
Median age (log)	− 1.726	− 2.200*	− 4.641***	− 6.927***	− 8.137***	− 7.092***	− 3.351**	− 7.572***	− 4.437**	− 2.092***
Unemployment rate (%)	− 0.009	0.146**	− 0.026	− 0.191**	− 0.189**	− 0.015	− 0.030	0.088	− 0.100	0.034
Family # unemployment rate (reference category)										
Skilled # unemployment rate	− 0.059	− 0.152***	− 0.102**	0.025	− 0.010	− 0.051	− 0.035	− 0.214***	− 0.073	− 0.018
Skilled regional # unemployment rate	− 0.009	− 0.209**	− 0.209**	− 0.012	na	na	na	na	0.066	− 0.096
Humanitarian # unemployment rate	− 0.027	− 0.090	0.395**	0.131	0.237**	0.286**	− 0.060	0.001	0.650**	0.022
% of compatriots (people born in the same country)	0.678***	0.312***	1.640***	2.309***	5.462***	4.027***	1.994	3.751***	5.018***	4.183***
Family # % compatriots (reference category)										
Skilled # % compatriots	0.089***	0.147***	0.710***	− 0.712***	− 1.469***	− 1.609**	3.441*	− 1.410***	0.562*	1.786***
Skilled regional # %compatriots	− 0.556***	− 0.382***	− 0.313	− 1.642***	na	na	na	na	− 4.211***	0.250
Humanitarian # % compatriots	− 0.392***	0.120*	− 0.506	0.311	− 2.079**	− 1.120*	0.076	− 0.619	− 3.340*	− 1.235
Constant	− 10.577	− 19.475**	8.965	19.276**	32.297***	15.010*	5.984	20.051**	11.613	− 5.379
Observations	348	348	348	348	261	261	261	261	348	348
Pseudo R-squared	0.131	0.155	0.137	0.145	0.132	0.118	0.0604	0.134	0.0604	0.136
Log Lik	− 2082	− 1974	− 1546	− 1610	− 1245	− 1256	− 1321	− 1024	− 1519	− 1511

Negative Binomial Regression, cluster by SA4 region robust error. *Source* ACMID 2016, Census 2011

***p < 0.01, **p < 0.05, *p < 0.1

While the majority of skilled migrants show a positive association with proportion of compatriots, skilled immigrants from Sri Lanka, Pakistan, Iran, and Egypt are more likely to reside in regions with a lower proportion of compatriots compared to family migrants from the same country of birth. Estimates for skilled regional migrants confirm that they are more likely to go to regions with a lower proportion of compatriots. This is the case for regional visa holders from India, China, Malaysia, Sri Lanka, and Nepal, but the opposite characterises migrants from Zimbabwe. The latter tend to be concentrated in regional areas of Western Australia, regardless of visa type. Humanitarian migrants from India, Malaysia, Pakistan, Iran, Egypt, Nepal, and Zimbabwe tend to move to regions with a lower proportion of compatriots compared to family migrants, and only humanitarian migrants from China, Sri Lanka and Thailand reside in regions with a higher proportion of people originating in the same country.

Regressions at the local (suburban) SA2 level allow us to closely study the existence of ethnic networks, which are best known to operate on this scale, whereas unemployment effects show best at the labour market level (SA4), which came through in our results from the regional level. Our regression estimates from SA2 level are presented in Table 5. All selected countries of birth show positive and significant association with proportion of compatriots. However, when broken down by visa categories, we notice predominantly negative association with proportion of compatriots among skilled migrants. Skilled immigrants from India, Sri Lanka, Pakistan, Iran, Nepal, Egypt, and Zimbabwe are less likely to locate in suburbs with a higher proportion of compatriots compared to family migrants. The opposite pattern characterises skilled visa holders from China, Malaysia, and Thailand, who are more likely to settle in suburbs with a higher proportion of compatriots than family migrants. Humanitarian migrants follow broadly the same pattern as skilled migrants and most of them tend to locate in suburbs with a lower proportion of compatriots. This perhaps reflects the current policy for humanitarian entrants who are preferably placed with family members already residing in Australia or are dispersed randomly across Australia with increasing flows to regional areas.

**Table 5:** Determinants of settlement choices of permanent migrants by country of birth in Australia in 2016 on SA2 level, regression estimates

Variables	India (4a)	China (4b)	Malaysia (4c)	Sri Lanka (4d)	Pakistan (4e)	Iran (4f)	Thailand (4g)	Egypt (4h)	Nepal (4i)	Zimbabwe (4j)
Population 2011 (log)	1.262***	1.454***	1.489***	1.260***	1.317***	1.601***	1.273***	1.660***	1.279***	1.307***
% of owned dwellings	− 0.010*	0.007	0.010	0.006	0.018***	− 0.007	0.003	0.015*	− 0.004	− 0.003
% of public housing	0.012	0.017	0.008	0.007	0.034	0.001	0.002	0.007	0.050*	− 0.023**
% of people with higher education (Bc and higher)	− 0.005	0.015**	0.015**	0.009	0.015***	0.021***	0.008*	0.013*	0.007	− 0.012**
Median weekly household income (log)	0.583*	0.972**	− 0.174	− 0.437	− 1.332***	0.115	− 0.982***	− 0.896*	− 0.946*	0.604
Median age (log)	− 2.664***	− 2.436***	− 4.147***	− 5.479***	− 5.247***	− 3.602***	− 3.061***	− 5.951***	− 4.612***	− 2.385***
Unemployment rate (%)	− 0.100***	0.057	− 0.005	− 0.078	− 0.044	0.045	− 0.068**	− 0.017	− 0.105**	0.017
Family # unemployment rate (reference category)										
Skilled # unemployment rate	− 0.003	− 0.120***	− 0.175***	− 0.065	− 0.091*	− 0.350***	− 0.116***	− 0.180***	− 0.083**	− 0.098***
Humanitarian # unemployment rate	0.218**	0.107	0.371***	0.197***	0.194***	0.131***	− 0.011	0.128**	0.383***	0.144**
% of compatriots (people born in the same country)	0.655***	0.377***	1.144***	2.378***	5.765***	2.671**	1.986***	5.477***	4.637***	5.014***
Family # % compatriots (reference category)										
Skilled # % compatriots	− 0.036	0.123***	0.203	− 0.794***	− 1.504***	− 2.749**	2.604***	− 2.431***	− 1.176*	− 0.407
Humanitarian # % compatriots	− 0.360***	0.097**	− 1.102***	− 0.681**	− 1.330**	− 0.716	2.956***	− 0.846	0.774	− 2.692***
Constant	− 3.176	− 10.426*	1.529	10.276**	14.255***	− 3.649	8.156*	9.081	11.029*	− 8.705*
Observations	6,336	6,336	6,336	6,336	6,336	6,336	6,336	6,336	6,336	6,336
Pseudo R-squared	0.169	0.153	0.133	0.131	0.109	0.0937	0.104	0.124	0.0989	0.143
Log Lik	− 17,243	− 17,325	− 10,293	− 10,075	− 9279	− 11,516	− 11,279	− 5552	− 7047	− 8038

Negative Binomial Regression, cluster by SA4 region robust error. *Source* ACMID 2016, Census 2011

***p < 0.01, **p < 0.05, *p < 0.1

All presented models and results are based on data from the latest census period 2011–2016. As a robustness check, we have replicated the analysis using data from the previous census period 2006–2011 by aggregating collection district data from the 2006 Census into SA2 and SA4 regions which were first introduced in the 2011 Census. Because of data sparsity, disaggregation by visa class and country of birth could be done at the SA2 level only for four countries out of the ten used in 2016 analysis, namely India, China, Sri Lanka, Thailand. Regression outputs are in Tables 7–10 of the Appendix. The results are consistent with the results from the 2016 analysis.

## Conclusion

With 28% of its residents born overseas, Australia is a high immigration country with a highly regulated visa policy that favours highly skilled migrants while facilitating family reunification and recognising the right of refuge. In this context, a growing body of work focussing on the settlement patterns of the overseas-born has demonstrated a strong preference toward major cities—particular in Sydney and Melbourne—and a preference for residential clustering with members of the same migrant group at a neighbourhood scale. While variations in settlement partners by country of birth and other socio-demographic attributes have been well researched (Guan, 2020; Hugo, 2011; Raymer et al., 2018; Wang et al., 2018; Wilson & Charles-Edwards, 2017), the role of visa class in shaping these patterns remains unclear. The recent release of ACMID, which links visa class with census data, provides a unique opportunity to address this gap for permanent migrants. Our analysis of ACMID data to understand settlement patterns by visa type has revealed three key findings.

First, compared to the Australia-born population, all migrants regardless of visa type are more likely to settle in regions with lower unemployment. Disaggregation by visa type shows that this association is most pronounced for skilled migrants, including those on regional visas. This suggests that the skilled migration program serves its key purpose of addressing labour shortages. By contrast, humanitarian visa holders are more likely to reside in regions and neighbourhoods with a higher unemployment rate, which raises concerns about their access to employment which is an integral part of building a new life in Australia. While this relationship does not hold at a labour market level, it is a source of concern because local unemployment is an indicator of deprivation that may be detrimental to socio-economic outcomes.

The second key finding is that skilled migrants generally do not respond to ethnic networks at the local level, except for skilled migrants from China, Malaysia and Thailand. Skilled migrants are considered economic migrants, they are expected to have more resources on arrival and to be less dependent on support from their compatriots. The case of Chinese, Malaysian and Thai skilled migrants suggests that there is some degree of residential sorting and voluntary preference for certain neighbourhood characteristics, which may include benefits derived from co-ethnic neighbours, or other attributes such as local schools, houses of worship, or a preference for a particular type of housing.

Finally, humanitarian migrants are more likely to reside in suburbs with a higher proportion of foreign-born but not necessarily from the same country of birth. This is a surprising result that does not align with evidence from other countries such as Sweden and the Netherlands, where there is growing residential segregation along ethnic lines. This could be explained by recent attempts of the Australian government to relocate humanitarian migrants in regional areas to alleviate population growth pressure from major urban centres. Conversely, some degree of preference could be involved, especially once factors such as housing affordability are considered. While being a part of a tight ethnic network in the long term might hinder spatial integration, ethnic networks offer much support in many areas of life during the initial phase of settlement or resettlement.

The current settlement patterns of immigrants are the result of both initial settlement upon arrival in Australia, and subsequent internal migration. Further research is required on how access to ethnic networks and higher unemployment may interact to affect future settlement and visa policies. We did not distinguish between these two processes in this paper because of data sparsity, which is particularly problematic when disaggregating migrant count data by country of birth and visa class at a suburb level. Individual-level approaches are a promising way forward to address this limitation thanks to new Australian datasets such as Multi-Agency Data Integration Project (MADIP), which combines longitudinal individual-level data on health, education, employment, income and taxation, government payments and population demographics, including migration. Using MADIP would permit establishing, for the first time in Australia, the retention and internal migration patterns of immigrants over time based on their visa status, while taking into account transitions from temporary to permanent residency.

## Figures and Tables

**Fig. 4:**
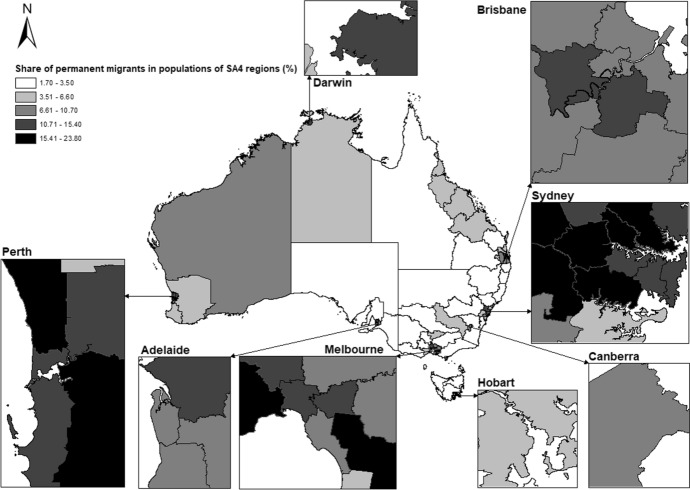
Share of permanent migrant populations of SA4 regions (%). *Source* Produced by author using ArcGIS

**Fig. 5:**
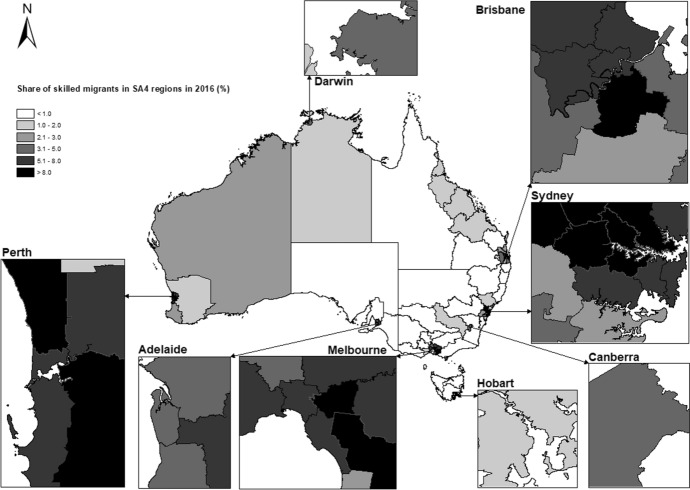
Share of skilled migrants in SA4 regions in 2016 (%). *Source* Produced by author using ArcGIS

**Fig. 6:**
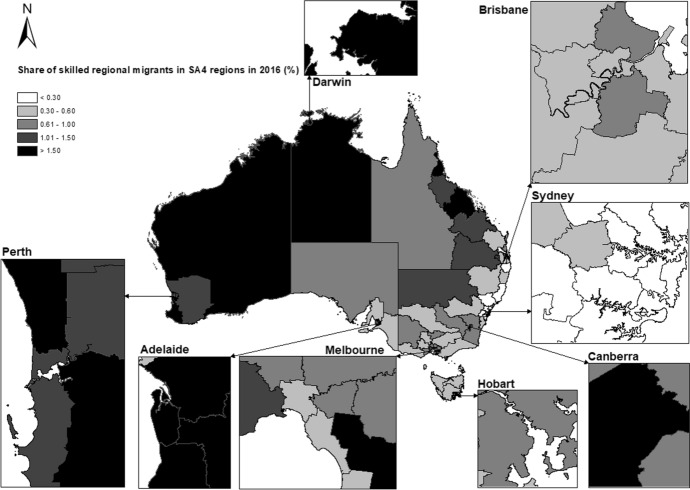
Share of skilled regional migrants in SA4 regions in 2016 (%). *Source* Produced by author using ArcGIS

**Fig. 7:**
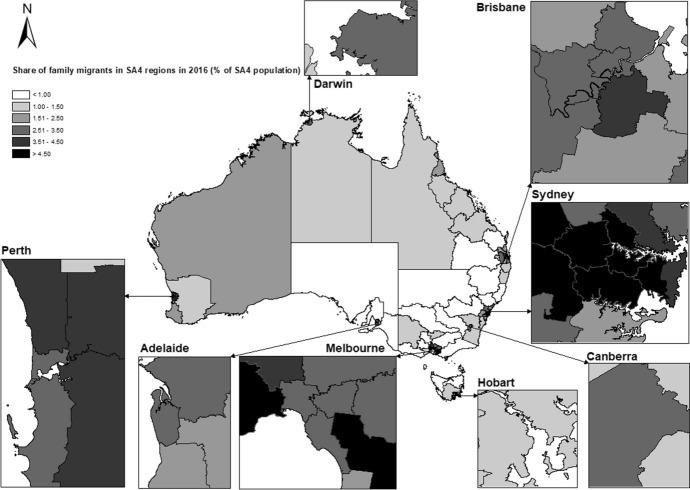
Share of family migrants in SA4 regions in 2016 (% of SA4 population). *Source* Produced by author using ArcGIS

**Fig. 8:**
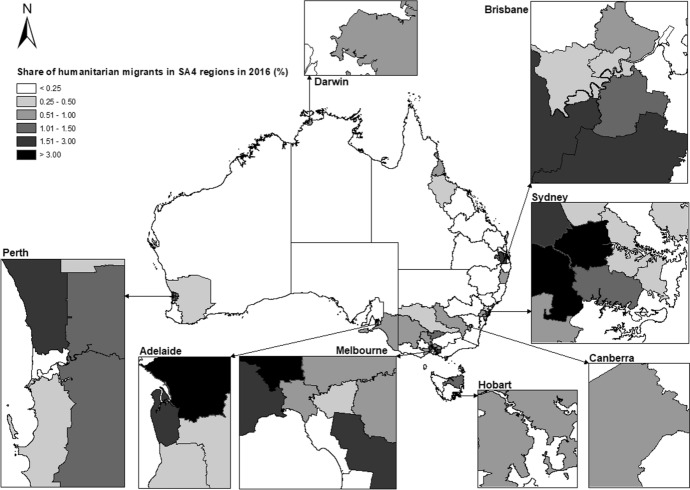
Share of humanitarian migrants in SA4 regions in 2016. *Source* Produced by author using ArcGIS

## Data Availability

All source data are publicly available through the Australian Bureau of Statistics.

## Appendix

See Figs. 4, 5, 6, 7, and 8; Tables 6, 7, 8, 9, and 10.

**Table 6:** Determinants of settlement choices of Australian-born and permanent migrants in Australia in 2016 on regional (SA4) and local (SA2) level, regression estimates

Variables	All permanent migrants	
	SA4 (1a)	SA2 (1b)
Population size in 2011 (log)	1.010***	0.982***
% of owned dwellings	0.003	− 0.000
% of public housing dwellings	− 0.006	− 0.002
% of people with higher education (bachelor and higher)	− 0.002	− 0.001
Median weekly household income (log)	0.293***	0.112
Median age (log)	− 0.820***	− 0.968***
Unemployment rate (%)	0.035***	− 0.008
Aus-born # unemployment rate (reference category)		
perm.migrants # unemployment rate	− 0.071***	− 0.050***
% of foreign-born	− 0.018***	− 0.018***
Aus-born # % foreign-born (reference category)		
perm.migrants # % foreign-born	0.076***	0.081***
Constant	0.533	3.053**
Observations	174	4,224
Pseudo R-squared	0.170	0.056
Log Lik	− 1806	− 32,964

Negative Binomial Regression, cluster by SA4 region robust error. *Source* ACMID 2016, Census 2011

***p < 0.01, **p < 0.05, *p < 0.1

**Table 7:** Determinants of settlement choices of Australian-born and permanent migrants in Australia in 2011 on regional (SA4) and local (SA2) level, regression estimates

Variables	All permanent migrants	
	SA4 (1a)	SA2 (1b)
Population size in 2006 (log)	1.026***	0.856***
% of owned dwellings	− 0.002	− 0.000
% of public housing dwellings	− 0.001	0.004
% of people with higher education (bachelor and higher)	− 0.004**	− 0.002
Median weekly household income (log)	0.037	0.315
Median age (log)	− 0.447	− 0.462*
Unemployment rate (%)	− 0.002	0.020
Aus-born # unemployment rate (reference category)		
perm.migrants # unemployment rate	− 0.113***	− 0.037***
% of foreign-born	− 0.015***	− 0.015***
Aus-born # % foreign-born (reference category)		
perm.migrants # % foreign-born	0.082***	0.090***
Constant	1.288	0.780
Observations	174	4164
Pseudo R-squared	0.180	0.148
Log Lik	− 1763	− 32,256

Negative Binomial Regression, cluster by SA4 region robust error. *Source* ACMID 2011, Census 2006

***p < 0.01, **p < 0.05, *p < 0.1

**Table 8:** Determinants of settlement choices of permanent migrants in Australia in 2011 on regional (SA4) and local (SA2) level, regression estimates

Variables	All permanent migrants	
	SA4 (2a)	SA2 (2b)
Population size in 2006 (log)	1.158***	0.797***
% of owned dwellings	0.005	− 0.001
% of public housing dwellings	0.044*	0.032***
% of people with higher education (bachelor and higher)	− 0.004	− 0.001
Median weekly household income (log)	− 1.149**	0.189
Median age (log)	− 2.123**	− 1.518***
Unemployment rate (%)	− 0.128***	− 0.007
Family # unemployment rate (reference category)		
Skilled # unemployment rate	− 0.146***	− 0.040**
Skilled regional # unemployment rate	− 0.184***	− 0.043
Humanitarian # unemployment rate	0.128*	0.162***
% of foreign-born	0.070***	0.063***
Family # % foreign-born (reference category)		
Skilled # % foreign-born	0.023***	0.033***
Skilled regional # % foreign-born	− 0.060***	− 0.025***
Humanitarian # % foreign-born	− 0.003	0.013
Constant	8.246	0.527
Observations	348	8,328
Pseudo R-squared	0.0896	0.0808
Log Lik	− 2879	− 42,327

Negative Binomial Regression, cluster by SA4 region robust error. *Source* ACMID 2011, Census 2006

***p < 0.01, **p < 0.05, *p < 0.1

**Table 9:** Determinants of settlement choices of permanent migrants by country of birth in Australia in 2011 on SA4 level, regression estimates

Variables	India (3a)	China (3b)	Malaysia (3c)	Sri Lanka (3d)	Pakistan (3e)	Iran (3f)	Thailand (3g)	Egypt (3h)	Nepal (3i)	Zimbabwe (3j)
Population 2006 (log)	1.476***	1.815***	1.540***	1.597***	1.753***	2.265***	1.321***	1.741***	1.247***	1.204***
% of owned dwellings	0.011	0.032***	0.032***	0.022**	0.043***	0.049***	− 0.006	0.079***	0.029*	− 0.015**
% of public housing	0.115***	0.139***	0.112**	0.077**	0.139***	0.170***	− 0.011	0.204***	0.173***	− 0.013
% of people with higher education (Bc and higher)	0.004	0.034***	0.005	0.012	0.021**	0.063***	0.000	0.045***	0.031**	− 0.017***
Median weekly household income (log)	− 0.338	0.671	− 0.491	− 1.643**	− 2.569***	− 1.556	− 0.780	− 3.178***	− 1.622*	− 0.143
Median age (log)	− 2.250	− 1.063	− 2.546	− 5.889***	− 5.717***	− 2.485	− 2.787	− 5.537***	− 5.247**	− 3.136***
Unemployment rate (%)	− 0.098	0.000	− 0.081	− 0.292***	− 0.228**	− 0.356***	− 0.110	− 0.152	− 0.251*	− 0.212***
Family # unemployment rate (reference category)										
Skilled # unemployment rate	− 0.106***	− 0.167***	− 0.092*	0.047	− 0.072	− 0.034	− 0.087	− 0.068	− 0.101	0.122*
Skilled regional # unemployment rate	− 0.044	− 0.185**	0.016	− 0.015	− 0.083	− 0.084	− 0.432***	0.178	− 0.193	0.002
Humanitarian # unemployment rate	− 0.138	− 0.039	0.181	− 0.077	0.093	0.229***	0.116	0.116	0.258	− 0.011
% of compatriots (people born in the same country)	1.193***	0.412***	1.519***	2.836***	11.380***	6.033***	2.255*	5.255***	23.360***	5.133***
Family # % compatriots (reference category)										
Skilled # % compatriots	0.050	0.160***	0.784***	− 0.669***	− 2.468**	− 3.457***	5.254**	− 2.382***	− 3.794	3.192***
Skilled Regional # %compatriots	− 0.897***	− 0.518***	0.272	− 1.931***	− 7.773***	− 9.369***	− 3.379*	− 2.844***	− 19.767***	− 0.468
Humanitarian # % compatriots	− 0.506**	0.296***	− 0.544	0.349	− 3.193	− 1.772**	− 1.566	-2.595**	− 24.655***	− 3.289**
Constant	− 4.276	− 21.550**	− 5.551	14.848*	16.243*	− 10.342	4.965	15.516	14.142	2.400
Observations	348	348	348	348	348	348	348	348	348	348
Pseudo R-squared	0.143	0.154	0.137	0.129	0.112	0.116	0.0732	0.105	0.0733	0.111
Log Lik	− 1816	− 1794	− 1355	− 1470	− 1226	− 1210	− 1337	− 1067	− 1024	− 1419

Negative Binomial Regression, cluster by SA4 region robust error. *Source* ACMID 2011, Census 2006

***p < 0.01, **p < 0.05, *p < 0.1

**Table 10:** Determinants of settlement choices of permanent migrants by country of birth in Australia in 2011 on SA2 level, regression estimates

Variables	India (4a)	China (4b)	Sri Lanka (4c)	Thailand (4d)
Population 2006 (log)	1.013***	1.454***	0.922***	1.332***
% of owned dwellings	− 0.013**	− 0.005	0.004	− 0.010*
% of public housing	0.021	0.024*	0.005	0.020
% of people with higher education (Bc and higher)	0.002	0.018***	0.021***	0.010*
Median weekly household income (log)	0.461	0.486	− 0.769	− 0.547
Median age (log)	− 2.776***	− 2.909***	− 4.963***	− 2.176***
Unemployment rate (%)	− 0.009	0.032	− 0.033	− 0.086
Family # unemployment rate (reference category)				
Skilled # unemployment rate	0.050	− 0.096***	0.057	− 0.021
Humanitarian # unemployment rate	0.174*	0.107**	0.185***	0.261***
% of compatriots (people born in the same country)	1.433***	0.583***	3.121***	2.095***
Family # % compatriots (reference category)				
Skilled # % compatriots	− 0.222***	0.158**	− 1.095***	3.663***
Humanitarian # % compatriots	− 0.878***	0.000	− 0.811	1.636
Constant	− 0.902	− 4.744	12.979**	1.722
Observations	6246	6246	6246	6246
Pseudo R-squared	0.157	0.151	0.110	0.0846
Log Lik	− 13,551	− 13,924	− 7940	− 8593

Negative Binomial Regression, cluster by SA4 region robust error. *Source* ACMID 2011, Census 2006

***p < 0.01, **p < 0.05, *p < 0.1

## Footnotes

1 From the complete SA4 structure we omitted 16 units—all the non-spatial special purpose zones, comprising Migratory–Offshore–Shipping and No Usual Address codes for each State and Territory. From the complete SA2 structure we omitted 84 units—all the ones with less than 10 residents (industrial zones or nature protected areas) and all non-spatial special purpose zones, comprising Migratory–Offshore–Shipping and No Usual Address codes for each State and Territory.

2 Index of Dissimilarity (I_D_).

$${I}_{D}=0.5{\sum }_{i=1}^{n}{|x}_{i}-{y}_{i}|$$

n = number of spatial units. ${x}_{i}$ = proportion of a particular migrant group living in region *i.* ${y}_{i}$ = proportion of the reference group (the Australian population here) in region *i.* I_D_ ranges from 0 to 100, 0 means complete integration (two populations have the same spatial distribution), 100 indicates complete segregation (no one from the migration group is in the same region as the reference population). I_D_ < 20—very little segregation, I_D_ > 30—significant segregation, I_D_ > 50—extreme segregation.
